# Selective activation of tumor growth-promoting Ca^2+ ^channel MS4A12 in colon cancer by caudal type homeobox transcription factor CDX2

**DOI:** 10.1186/1476-4598-8-77

**Published:** 2009-09-25

**Authors:** Michael Koslowski, Özlem Türeci, Christoph Huber, Ugur Sahin

**Affiliations:** 1 Department of Internal Medicine III, Experimental and Translational Oncology, Johannes Gutenberg University, Obere Zahlbacherstr. 63, 55131 Mainz, Germany; 2 Ganymed Pharmaceuticals AG, Freiligrathstr. 12, 55131 Mainz, Germany

## Abstract

Colon cancer-associated MS4A12 is a novel colon-specific component of store-operated Ca^2+ ^(SOC) entry sensitizing cells for epidermal growth factor (EGF)-mediated effects on proliferation and chemotaxis. In the present study, we investigated regulation of the *MS4A12 *promoter to understand the mechanisms responsible for strict transcriptional restriction of this gene to the colonic epithelial cell lineage. DNA-binding assays and luciferase reporter assays showed that *MS4A12 *promoter activity is governed by a single CDX homeobox transcription factor binding element. RNA interference (RNAi)-mediated silencing of intestine-specific transcription factors CDX1 and CDX2 and chromatin immunoprecipitation (ChIP) in LoVo and SW48 colon cancer cells revealed that *MS4A12 *transcript and protein expression is essentially dependent on the presence of endogenous CDX2. In summary, our findings provide a rationale for colon-specific expression of *MS4A12*. Moreover, this is the first report establishing CDX2 as transactivator of tumor growth-promoting gene expression in colon cancer, adding to untangle the complex and conflicting biological functions of CDX2 in colon cancer and supporting MS4A12 as important factor for normal colonic development as well as for the biology and treatment of colon cancer.

## Findings

Membrane-spanning 4-domains subfamily A (MS4A) is an evolving family of structurally related cell surface proteins. Prominent members of this group are B lymphocyte differentiation antigen CD20 (MS4A1), the high-affinity IgE receptor β chain (FcεRIβ; MS4A2), and hematopoietic cell cycle regulator HTm4 (MS4A3) [1-3]. As reported recently, a genome-wide in silico search for differentiation genes of colonic epithelial cells led us to *MS4A12*, a poorly characterized member of this family [4]. In contrast to other MS4A proteins, which are known to be restricted to cells of hematopoietic or lymphatic lineages, MS4A12 is not expressed in such cell types [2,3]. Instead, we showed that expression of MS4A12 is strictly confined to the apical region of normal colonic epithelial cells. In the course of malignant transformation expression of MS4A12 is frequently and strongly maintained in colon cancers. Functional studies revealed that MS4A12 is a SOC channel modulating growth factor-mediated capacitative Ca^2+ ^entry, which is a convergent point of many signal transduction pathways controlling important cellular functions. In line with these findings, silencing of *MS4A12 *expression in colon cancer cells by RNAi results in attenuation of EGF-dependent effects. In particular, proliferation, motility, and chemotactic invasion of cells are significantly impaired. Cancer cells expressing MS4A12, in contrast, are sensitized to EGF and respond to low concentrations of this growth factor [4].

The molecular mechanisms regulating tissue-specific expression of *MS4A12 *have not been specified so far, however, identification of these mechanisms is likely to further shed light on the biological functions of MS4A12, its role in colon cancer progression, and its suitability as therapeutic molecular target. Therefore, we now aimed to identify the cis-elements and trans-factors that regulate *MS4A12 *promoter activity.

**Figure 1:**
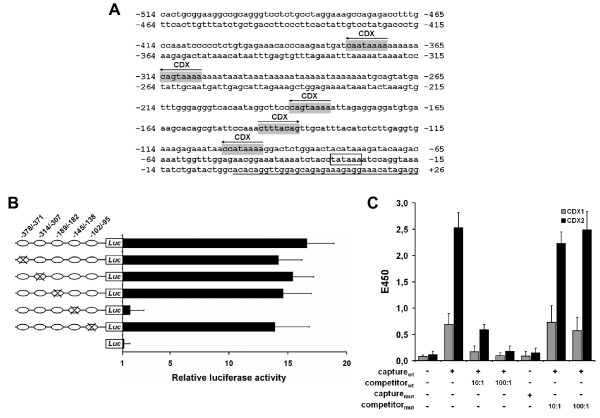
***MS4A12 *promoter activity is governed by a single functional CDX binding element**. (**A**) Proximal 5'-flanking region of the *MS4A12 *gene. Predicted CDX binding sites are shown in gray, orientation of the elements is depicted by arrows. The TATA box element is boxed, transcription start is underlined. (**B**) Activation of the *MS4A12 *promoter measured in luciferase activity assays. The *MS4A12 *promoter region (-952 to +12) was cloned into the basic pGL4 luciferase reporter vector (Promega). Variants containing mutated CDX binding sites were generated using QuickChange Site-Directed Mutagenesis Kit (Stratagene). 4 × 10^5 ^cells were seeded into 6-well plates and transfected with promoter constructs using Lipofectamine (Invitrogen). 24 h after transfection luciferase activity was measured in cell lysates and normalized to fluorescence obtained by co-transfection with eGFP reporter plasmid. All assays were done in triplicates; mean values +/- STD are shown. (**C**) Binding of CDX1 and CDX2 to the predicted binding site was assessed using NoShift Transcription Factor Assay Kit (Novagen) following the manufacturer's instructions. Biotinylated double-stranded oligonucleotides featuring the wild type (capture_wt_) (5'-cgt att cca aac ttt aca gtt gca ttt ac-3') or the mutated (capture_mut_) (5'-cgt att cca aac CCC aca gtt gca ttt ac-3') CDX consensus element in the *MS4A12 *promoter binding site, and non-biotinylated wild type (competitor_wt_) or mutated (competitor_mut_) oligonucleotides, were incubated with nuclear extracts of LoVo cells, transferred to a streptavidin-coated 96-well plate and incubated for 1 h at 37°C. Antibodies specific for CDX1 and CDX2 (both from Abcam) were added to the samples and incubated for 1 h at 37°C. The secondary horseradish peroxidase-conjugated antibody was incubated for 30 min at 37°C. TMB substrate was added to the samples to develop colorimetric signals. The reaction was quenched with 1 N HCl and sample absorbance was read at 450 nm on a Wallac Victor^2 ^multi-label counter (Perkin Elmer).

As a first step for exploration of *MS4A12 *gene regulation, we analyzed the sequence of the *MS4A12 *promoter region using TFsearch program  and TRANSFAC database . This led to the identification of a typical TATA box element (TATAAA) at position -31 to -26 and several other putative cis-acting elements (Fig. 1a). Prediction of five CDX binding sites resembling the consensus sequence 5'-YTTTAYNR-3' was of particular interest, because the caudal-related proteins CDX1 and CDX2 are important regulators of intestinal development due to their specific transactivation of intestinal gene expression [5]. Four of the predicted CDX binding sites (positions -378 to -371, -314 to -307, -189 to -182, and -102 to -95) were in antisense (-) orientation, whereas one sequence element (-145 to -138) was in sense (+) orientation. To analyze whether expression of *MS4A12 *is under control of CDX transcription factors, we expressed luciferase reporter genes directed by the wild type *MS4A12 *promoter domain (-952 to +12) or by five promoter variants, each mutated at one of the putative CDX binding sites, in MS4A12-positive LoVo colon cancer cells. No reduction of promoter activity was observed by mutation of the elements in antisense (-) orientation as compared to wild type control. In contrast, mutation of the sense (+) orientated element at position -145 to -138 completely abolished *MS4A12 *promoter activity (Fig. 1b), suggesting exclusive relevance of this element for transcriptional regulation of *MS4A12*.

Next, to determine whether CDX1 and CDX2 were able to bind to this functional CDX element, an ELISA-based transcription factor binding assay was performed. Incubation of nuclear cell extract from LoVo cells as source of CDX transcription factors with a biotinylated oligonucleotide capture probe representing the putative CDX binding sequence resulted in detection of specific CDX1 and CDX2 protein-DNA complexes (Fig. 1c), albeit signals were much stronger for CDX2 compared to CDX1. Mutation of the biotinylated oligonucleotide probe suppressed binding of both transcription factors. Complex formation was also inhibited by excess amounts of the non-biotinylated capture probe as competitor, but not by a competitor probe with a mutated binding site, confirming specificity of these interactions. Due to the highly conserved homeodomain, CDX1 and CDX2 typically bind and transactivate many of the same DNA elements, but there are reports of gene promoters in which selective binding of CDX2 was observed [6-9]. In accordance with such reports, our data imply preferential binding of CDX2 to the functional element.

**Figure 2:**
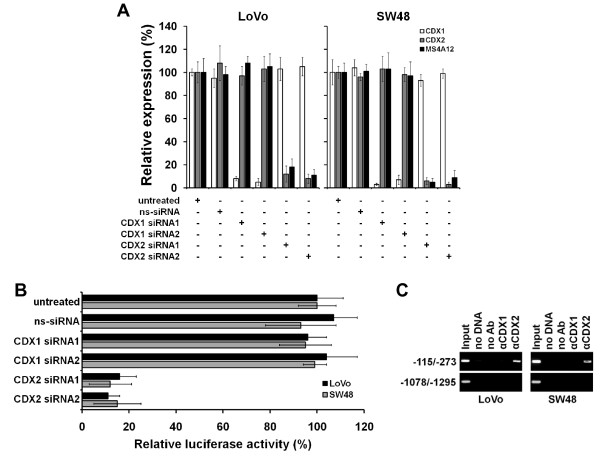
**Selective activation of *MS4A12 *by CDX2**. (**A**) mRNA expression of *CDX1*, *CDX2*, and *MS4A12 *was quantified by real-time RT-PCR 48 h after transfection of LoVo and SW48 cells with 10 nM siRNA duplexes (Qiagen) using HiPerFect transfection reagent (Qiagen). siRNA duplexes targeted nucleotides 1054-1072 (siRNA1 sense 5'-r(CAG UAA GCC UGU UGG AUA A)dAdG-3', antisense 5'-r(UUA UCC AAC AGG CUU ACU G)dCdA-3') and 1675-1693 (siRNA2 sense 5'-r(GGA UGC AGC UUC AAG AAU A)dAdA-3', antisense 5'-r(UAU UCU UGA AGC UGC AUC C)dAdA-3') of the *CDX1 *mRNA sequence (NM_001804.2), and nucleotides 1492-1510 (siRNA1 sense 5'-r(GAG AGG GAC UCA AGG GAA A)dGdG-3', antisense 5'-r(UUU CCC UUG AGU CCC TCU C)dTdC-3') and 1263-1281 (siRNA2 sense 5'-r(GAG UAA GAC AAG UGG GAU U)dTdC-3', antisense 5'-r(AAU CCC ACU UGU CUU ACU C)dCdT-3') of the *CDX2 *mRNA sequence (NM_001265.2). As control a non-silencing (ns) siRNA duplex (sense 5'-r(UAA CUG UAU AAU CGA CUA G)dTdT-5', antisense 5'-r(CUA GUC GAU UAU ACA GUU A)dGdA-3') was used. RNA extraction, first-strand cDNA synthesis and real-time reverse transcription-PCR (RT-PCR) were performed as previously described [4]. Real-time quantitative expression analysis was performed in triplicates in a 40 cycle RT-PCR. After normalization to *HPRT *(sense 5'-TGA CAC TGG CAA AAC AAT GCA-3'; antisense 5'-GGT CCT TTT CAC CAG CAA GCT-3', 62°C annealing) expression of *CDX1 *(sense 5'-CTC ACT GAA CGG CAG GTG AAG-3'; antisense 5'-TAG GTG ACT GTC CAC CAT GTC-3', 60°C annealing), *CDX2 *(sense 5'- GCT TCT GGG CTG CTG CAA ACG-3'; antisense 5'-CTT TCG TCC TGG TGG TTT TCA CTT GG-3', 62°C annealing), and *MS4A12 *(sense 5'- GAG CTT TCC CGT TGT CTG GTG-3'; antisense 5'- GCT GAA GAA GAC GCT GGT GTC-3', 60°C annealing) was quantified using ΔΔCt calculation. Expression of each gene is shown in relation to expression levels in untreated control cells (100%). (**B**) 48 h after transfection with siRNA duplexes LoVo and SW48 cells were transfected with luciferase reporter genes linked to the wild type *MS4A12 *promoter domain (-952 to +12). Activation of the *MS4A12 *promoter was measured in luciferase activity assays after 24 h. Luciferase activity was normalized to fluorescence obtained by co-transfection with eGFP reporter plasmid. All assays were done in triplicates; mean values +/- STD are shown. (**C**) ChIP was performed using the Chromatin Immunoprecipitation (ChIP) Assay Kit (Millipore) according to the manufacturer's instructions with chromatin prepared from LoVo and SW48 cells. The promoter region containing the CDX2 binding site (-115/-273) and a region upstream to the promoter (-1078/-1295) as negative control were analyzed by PCR following immunoprecipitation with the antibodies indicated. Results of amplification of soluble chromatin prior to precipitation are shown as control (input).

To directly assess the impact of CDX1 and CDX2 on MS4A12 transcript and protein expression we silenced both factors in LoVo and SW48 colon cancer cells by transient transfection with specific siRNA duplexes. Transcript levels of CDX1 and CDX2 were specifically reduced by >90% compared to non-transfected cells and cells transfected with non-silencing control siRNA duplexes (Fig. 2a). Strikingly, only silencing of CDX2 resulted in concomitant loss of *MS4A12 *mRNA expression. Luciferase reporter assays, showing selective impairment of *MS4A12 *promoter activity in both cell lines upon transfection with CDX2-specific but not with CDX1-specific siRNA duplexes reinforced lack of promoter transactivation as underlying mechanism for the loss of *MS4A12 *expression (Fig. 2b). Accordingly, selective binding of CDX2 to the endogenous *MS4A12 *promoter was shown by ChIP assays in LoVo and SW48 cells (Fig. 2c).

**Figure 3:**
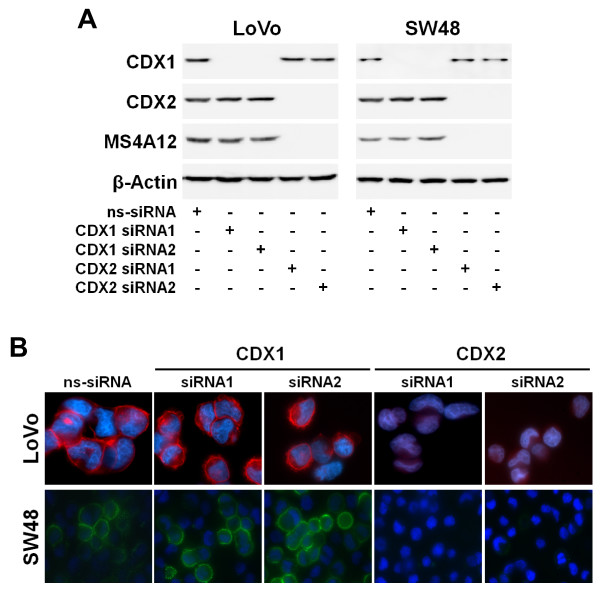
**Expression of MS4A12 protein is dependent on endogenous CDX2**. (A) Protein expression of CDX1, CDX2, and MS4A12 in LoVo and SW48 cells analyzed by Western blot 48 h after transfection with siRNA duplexes. Whole cell lysates were prepared using RIPA lysis buffer. Extracts were diluted in reducing sample buffer (Roth), subjected to SDS-PAGE and subsequently blotted onto PVDF membrane (Pall). Immunostaining was performed with an antibody reactive to MS4A12 [4], CDX1, CDX2, and beta-Actin (all from Abcam), followed by detection of primary antibodies with horseradish peroxidase-conjugated secondary antibodies (Jackson ImmunoResearch Laboratories). β-Actin was used as loading control. (**B**) Immunofluorescence analysis of MS4A12 expression in LoVo and SW48 cells 48 h after transfection with siRNA duplexes using an antibody reactive to MS4A12 [4] and secondary antibodies conjugated with Alexa Fluor 546 (LoVo) or Alexa Fluor 488 (SW48).

The selective action of CDX2 was confirmed on protein level by Western blot analyses, as MS4A12 protein expression was unaffected by CDX1 silencing, whereas no MS4A12 protein could be detected in cells after CDX2 silencing (Fig. 3a). Consistently, distinct plasma membrane staining of LoVo and SW48 cells with a polyclonal anti-MS4A12 antibody was lost upon siRNA-induced silencing of CDX2 but not after silencing of CDX1 (Fig. 3b). In summary, these investigations disclosed that expression of *MS4A12 *is selectively activated by CDX2.

Our findings are of particular interest for several reasons. First, they provide a rationale for the strict transcriptional restriction of *MS4A12 *to the colonic epithelium and its absence from other normal cell types. In the adult intestine CDX2 expression increases progressively from the duodenum to the distal intestine, and is at its greatest levels in the proximal colonic epithelium [10,11]. CDX2 target genes are expressed in a number of different patterns in the small intestine and colonic epithelium. These include genes like *mucin 2 (MUC2)*, *Kruppel-like factor 4 (KLF4)*, and *guanylcyclase 2C (GUCY2C)*, which are expressed throughout the intestine [9,12,13]. The observation that *MS4A12 *expression is strictly confined to the colonic epithelial cells is concordant with other CDX2 target genes showing colon-restricted expression, like colon-specific *carbonic anhydrase I (CA1) *[14]. Precise temporal and spatial regulation of gene expression within the intestinal and colonic epithelium are known to depend upon specific interactions between CDX2 and other transcription factors [15-18], as well as the phosphorylation state of CDX2 [19,20], which will be mapped for *MS4A12 *in future studies. In colon cancer cells CDX2 expression was initially reported to be reduced compared to normal colonic mucosa with an inverse relationship between CDX2 expression and advanced stages of cancers [21-24]. These data, however, have been disproved by recent studies showing strong and robust expression of CDX2 in >80% of colon cancers [25-27], complying with the high and frequent expression of *MS4A12 *in colon cancer.

Second, our data add to untangle the complex and conflicting biological functions of CDX2 in colon cancer. CDX2 has been reported to possess growth-inhibitory as well as growth-promoting functions in cancer cells. The suppressive function of CDX2 on tumor cell proliferation observed in several studies [28-30] has been explained by CDX2-mediated activation of negative cell cycle regulators, such as cyclin-dependent kinase inhibitor 1A (CDKN1A), a known inhibitor of G1 cyclin/cyclin-dependent kinase complexes [6]. On the other hand, it was shown that CDX2 enhances proliferation and has tumorigenic potential in the human colon cancer cell lines LoVo and SW48 [12] used in this study. Until now the molecular basis for the growth-promoting functions of CDX2 in these cells has not been elucidated. Together these conflicting findings point to a complex role for CDX2 in the regulation of cell proliferation. It suggests that CDX2 has different functions depending on the cellular and tissue context it is expressed in.

Our findings provide first evidence for the capability of this caudal-related protein to induce growth-promoting genes in colon cancer. Moreover, they link CDX2 to EGF-mediated cancer cell proliferation, migration and invasion, and provide a basis to further dissect the role of CDX2 in colon cancer progression.

## Conflicts of interests

MK is scientific advisor, ÖT is CEO/CSO, CH is member of the supervisory board, and US is CMO of Ganymed Parmaceuticals AG, a company holding patent applications on MS4A12 as therapeutic target for monoclonal antibody therapy of cancer.

## Authors' contributions

MK designed the study, carried out the experiments and drafted the manuscript. ÖT, CH, and US helped to design the study and have critically revised the manuscript draft. All authors have read and approved the final manuscript.
